# Ball milling induced borophene flakes fabrication†

**DOI:** 10.1039/d3ra02400h

**Published:** 2023-06-05

**Authors:** Klaudia Zielinkiewicz, Daria Baranowska, Ewa Mijowska

**Affiliations:** a Department of Nanomaterials Physicochemistry, Faculty of Chemical Technology and Engineering, West Pomeranian University of Technology Piastow Ave. 42 71-065 Szczecin Poland zk43130@zut.edu.pl emijowska@zut.edu.pl

## Abstract

To fill the knowledge gap for borophene, as the youngest member of the two-dimensional (2D) nanomaterials family, a facile, cost effective, scalable and reproducible fabrication route is still strongly required. Among so far studied techniques the potential of pure mechanical processes such as ball milling is not explored yet. Therefore, in this contribution, we explore the efficiency to exfoliate bulk boron into a few-layered borophene induced by mechanical energy in the planetary ball mill. It was revealed that the resulting flakes thickness and distribution can be controlled by (i) rotation speed (250–650 rpm), (ii) time of ball-milling (1–12 hours), and mass loading of bulk boron (1–3 g). Furthermore, the optimal conditions for the ball-milling process to induce efficient mechanical exfoliation of boron were determined to be 450 rpm, 6 hours, and 1 g (450 rpm_6 h_1 g), which resulted in the fabrication of regular and thin few-layered borophene flakes (∼5.5 nm). What is more, the mechanical energy induced during ball-milling, and the heat generated inside, affected the structure of borophene resulting in different crystalline phases. Besides being an additional and interesting discovery, it will also open up opportunities to investigate the relevance between the properties and the emerging phase. Structures labeled as β-rhombohedral, γ-orthorhombic, τ-B and the conditions under which they appear, have been described. Therefore, in our study, we open a new door to obtain a bulk quantity of few-layered borophene for further fundamental studies and practical potential assessment.

## Abstract 2

The preparation of borophene flakes by ball milling is shown in the following schematic.

## Graphical abstract

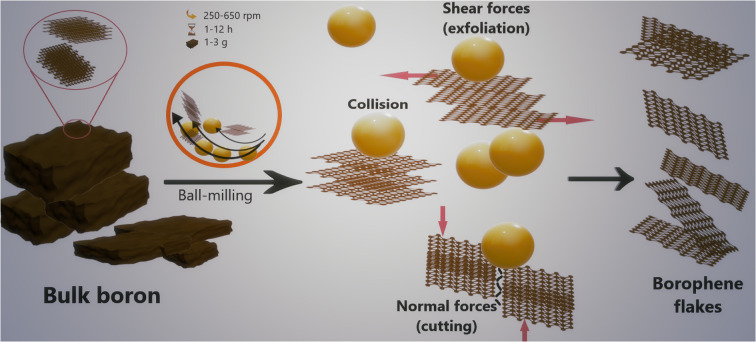

## 1. Introduction

Borophene, a single-atom thick sheet of boron, is now considered as one of the most enigmatic members in the world of two-dimensional (2D) materials, due to its structural and electronic properties which still need to be deeply explored.^1,2^ It exhibits various crystal structures and allotropes with different properties.^3^ All known boron structures contain B12 icosahedral clusters. The most common are α-rhombohedral, β-rhombohedral, α-tetragonal, β-tetragonal, YB66, orthorhombic or amorphous boron.^4^ The appearance of different structures and the disclosure of their allotropes is influenced by the use of different methods/parameters of synthesis.^5^ Envisioned by theorists^6,7^ borophene is known for its high-temperature superconductivity,^8^ metallicity,^9^ excellent elastic strength, and electronic mobility,^10^ making it a promising material for different applications such as an anode material in more powerful lithium-ion batteries,^11^ catalytic capabilities,^12,13^ and sensors.^14,15^ The scientific community is excited about the extraordinary range of potential applications that borophene can be used for. Like other 2D materials, borophene has potential applications in electronics,^16^ photonics,^17^ biomedical technologies,^18^ energy conversion, and storage.^19^

To date, some methods of borophene fabrication including top-down and bottom-up approaches have been reported.^5^ For example, Zhang *et al.*^20^ investigated which solvent among acetone, *N*,*N*-dimethyl formamide (DMF), acetonitrile, ethanol, and *N*-methyl pyrrolidone (NMP), is the most suitable for exfoliation of boron in a solvothermal-assisted liquid phase process. The resulting borophene with few layers was obtained in the assistance of acetone. Chowdhury *et al.*^21^ proposed an electrochemical exploration process, where boron was used as a cathode material. The borophene lateral particle size achieved, in this approach, was 400 to 600 nm. Similarly, Sielicki *et al.*^22^ proposed electrochemical exfoliation of the boron with the use of Cu/Ni meshes in Li^+^/DMSO and SO_4_^2−^/H_2_O solutions to create a new route of few-layered borophene (the thickness of obtained borophene was 3–6 nm). All the above-mentioned techniques have limitations for large-scale borophene production such as complexity and high cost.

On the other hand, the ball-milling process is now on the cusp of delivering thin layers of 2D materials. For example, Namba *et al.*^23^ prepared thin hexagonal boron nitride (h-BN) flakes. The research shows that the ball-milling process exfoliates the bulk of pristine h-BN. Zhang *et al.*^24^ synthesized a few-layered Ti_3_C_2_TX (MXene) nanodot composite *via* this method. The obtained nanodots are 2–5 nm in thickness and 6 nm in length. Tayyebi *et al.*^25^ have demonstrated bulk MoS_2_ exfoliation by ball-milling and gained nanosheet in high yield. However, there is a knowledge gap about the application of a ball mill in the exfoliation of bulk boron. This facile, low cost and scalable approach would allow a better understanding of the structural properties and performance of borophene.

Therefore, in this study, we filled this gap in the current state of the art by investigating the influence of ball-milling on the efficiency of few-layered borophene fabrication. The morphology of borophene was controlled by adjusting several factors, including rotation speed (250, 450, 650 rpm), ball-milling time (1, 3, 6, 12 h), and mass loading of boron (1, 2, 3 g). Detailed analyses based on microscopic and spectroscopic techniques have been conducted showing that the exfoliation process is assisted by the phase transition during ball milling, induced by mechanical energy, in the resulting sample of borophene. Therefore, here we propose a general route to generate the bulk quantity of few-layered borophene for exploration of the properties and application of this 2D nanomaterial.

## 2. Experimental section

### 2.1 Materials

Boron powder was purchased from Sigma Aldrich (USA).

### 2.2 Mechanochemical exfoliation

The ball-milling process was carried out for the preparation of few-layered borophene using a planetary zirconia ball mill from Fritsch Pulverisette. The operational parameters of the ball-milling process included rotation speed (250, 450, and 650 rpm), time of ball-milling (1, 3, 6, and 12 h), and the mass loading of starting bulk boron (1, 2, 3 g). Each modification was performed with the same number of zirconia balls (18). Moreover, to prevent the ball mill from heat exposure the experiments were carried out by 30 minutes cycles with 10 minutes breaks between each cycle. The obtained samples were labeled according to conducted parameters in order of rotation speed_time of ball-milling_quantity of boron, for example, 450 rpm_6 h_1 g means 450 rpm, 6 h of ball-milling, and 1 g of loaded boron.

## 3. Characterization

Determination of the thickness and lateral size of the borophene flakes was controlled by Atomic Force Microscopy (AFM) using Nanoscope V Multimode 8 Bruker. The crystal structure of the studied materials was investigated by X-ray Powder Diffraction (XRD) utilizing an Aeris, Malvern Panalytical apparatus employing CuKα radiation. The morphology of the obtained materials was examined with the use of Transmission Electron Microscopy (TEM) utilizing the FEI Tecnai F20 microscope coupled with an energy dispersive X-ray spectrometer (EDX) and Scanning Electron Microscopy (SEM; VEGA3 TESCAN, Brno, Czech Republic) equipped with a spectroscopic analysis modulus—X-ray energy dispersive (Bruker, Billerica, MA, USA). Fourier Transform Infrared Reflection (FTIR) spectra were acquired on a Nicolet 6700 FT-IR spectrometer. Obtained materials were mixed with KBr powder to create pellets. Additionally, sample 450 rpm_6 h_1 g was examined by X-ray Photoelectron Spectroscopy (XPS) using a Prevac system with a Scienta SES 2002 electron energy analyzer employing MgKα radiation.

## 4. Results and discussion

**Fig. 1:**
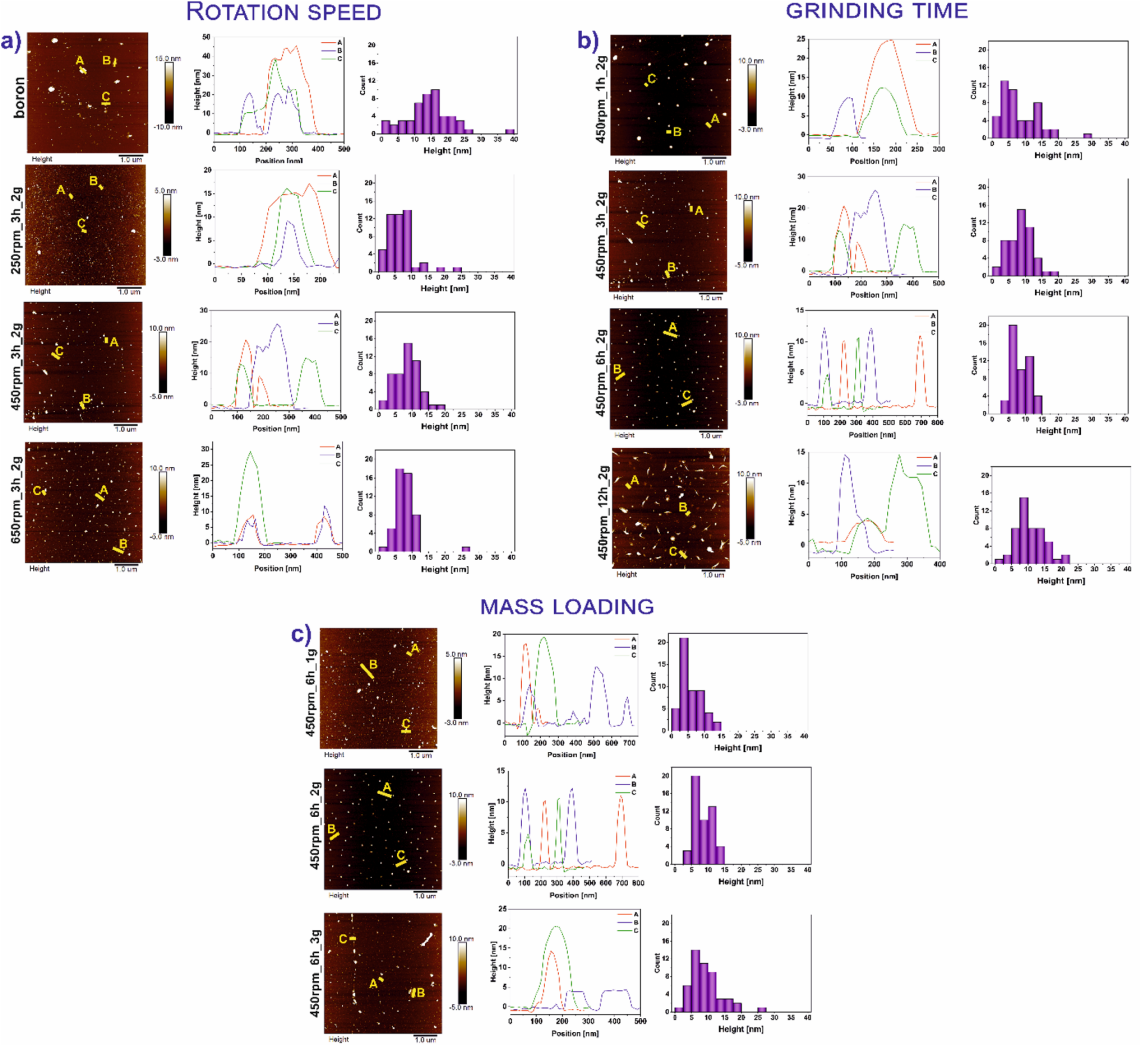
Atomic force microscopy (AFM) images of all studied materials (left panel) and height profiles of representative borophene flakes named A, B, C (middle panel) and corresponding histograms presenting thickness distribution of respective sample (right panel) in different operating parameters such as: (a) rotation speed [bulk boron, 250 rpm_3 h_2 g, 450 rpm_3 h_2 g, 650 rpm_3 h_2 g, (b) grinding time [450 rpm_1 h_2 g, 450 rpm_3 h_2 g, 450 rpm_6 h_2 g, 450 rpm_12 h_2 g] and (c) mass loading [450 rpm_6 h_1 g, 450 rpm_6 h_2 g, 450 rpm_6 h_3 g].

**Fig. 2:**
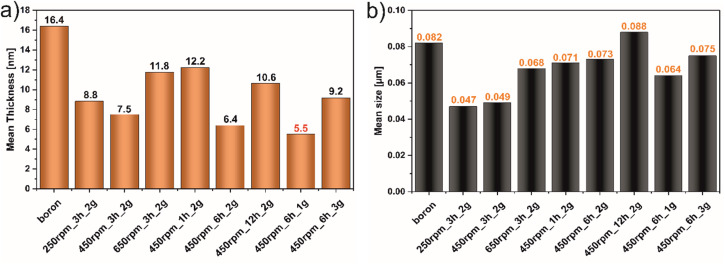
Statistical distribution of (a) mean thickness and (b) mean size of the samples.

Fig. 1 demonstrates the AFM images of all studied materials (left panel) and height profiles of representative borophene flakes named A, B, C (middle panel) and corresponding histograms presenting thickness distribution of respective sample (right panel). To be more specific, Fig. 1a represents the results of the samples tuned by rotation speed, Fig. 1b shows the data of the materials modified upon grinding time and Fig. 1c depicts the results obtained with different mass loading of bulk boron. The mean height of bulk boron is ∼16.4 nm. The reduction of the mean height of flakes has occurred upon ball-milling operating at different rotation speeds exhibited in Fig. 1a: for 250 rpm_3 h_2 g (∼8.8 nm), and 450 rpm_3 h_2 g (∼7.5 nm). An increase in the speed up to 650 rpm (650 rpm_3 h_2 g) causes the reagglomeration of some of the borophene (∼11.8 nm). Considering the rotation speed as a main factor for borophene fabrication, the speed of 450 revolutions per minute was chosen as a constant value for further investigations. A. Nugriho *et al.*^26^ described that rotation speed has a significant role in generating the mode motion of the ball. In this process, they have distinguished six modes labeled slipping, slumping, rolling, cascading, cataracts, and centrifuging.^27^ To be more specific, the slipping mode takes place when the rotation speed is low, while the centrifuging mode is at a high rotation speed. In our work grinding mode was activated at 450 rpm due to the formation of the thinnest flakes in this condition. Next, the dependence of flakes thickness on grinding time has been shown in Fig. 1b. A significant decrement in the bulk material was observed comparing the thickness of samples 450 rpm_1 h_2 g (∼12.2 nm) and 450 rpm_3 h_2 g (∼7.5 nm). The thickness of 450 rpm_6 h_2 g was ∼6.4 nm, while the sample 450 rpm_12 h_2 g showed a value of ∼10.6 nm which is close to the reference material. These data suggest that both, short (1 h) and long (12 h) time of ball-milling, cause no apparent effect on exfoliation of boron. A short time of ball-milling has a weak impact on the mechanical exfoliation process, meanwhile, a long time of ball-milling contributes to the reagglomeration of flakes, and hence an increase in thickness has been noticed. Therefore, 6 hours have been adopted as the optimal ball-milling time. Finally, concerned the mass loading of the precursor bulk boron equal to 1, 2 or 3 g has been evaluated and data are presented in Fig. 1c. The mean flakes heights of 450 rpm_6 h_1 g, 450 rpm_6 h_2 g, and 450 rpm_6 h_3 g were ∼5.5, ∼6.4, and ∼9.2 nm, respectively. The thinnest flakes of borophene were obtained at 450 rpm_6 h_1 g (∼5.5 nm). Therefore, it is clear that considering Ball to Powder Weight Ratio (BPR) induces a crucial effect on reducing particle size.^28^ This parameter describes the amount of energy delivered to the system concerning the used input of bulk material. The decreased initial mass of powder leads to an increase in the number of collisions per time unit and therefore the shock frequency, leading to efficient particle size reductions.^29^ The same effect was seen in our experiments when boron loading was reduced to 1 g. According to the literature,^30^ the single borophene sheet is 0.8 nm, thus the 450 rpm_6 h_1 g is composed of approximately 6 layers. For summary, Fig. 2a shows the statistical distribution of mean thickness, while Fig. 2b presents the mean lateral size of all studied samples (located in the range of 47–88 nm). What is more, the lateral size of all few-layered borophene flakes after the ball-milling process, excluding the sample 450 rpm_12 h_2 g, is smaller than bulk boron, proving that this strategy is a sufficient way to reduce the lateral size distribution. The 450 rpm_12 h_2 g has the highest value of lateral size, even higher than starting boron, which also confirms that 12 hours of ball-milling contribute to the reagglomeration of borophene flakes, resulting in bigger and thicker borophene flakes. The increase in particle size has been observed in two processes related to milling time: (i) during grinding for a short time, when weak and reversible aggregation occurs due to van der Waals forces, and (ii) for too long time, when reagglomeration occurs.^31^ Moreover, the obtained results related to dimensions such as thickness and lateral sizes are summarized in Table 1. Additional experiments to fabricate borophene flakes which were carried out at an intermediate value between 450 rpm and 650 rpm, also using 0.5 and 0.25 g of bulk boron under optimal operating parameters (450 rpm and 6 h) are attached in ESI (Fig. S1–4).†

**Table 1:** The statistical distribution of the thickness and lateral size of boron and borophene flakes

	Sample	Thickness [nm]	Lateral size [μm]
	Boron	0.025–39.034	0.030–0.261
Rotation speed	250 rpm_3 h_2 g	0.999–23.188	0.021–0.118
	450 rpm_3 h_2 g	0.410–18.795	0.021–0.090
	650 rpm_3 h_2 g	1.453–25.879	0.020–0.137
Grinding time	450 rpm_1 h_2 g	0.559–29.180	0.030–0.170
	450 rpm_3 h_2 g	0.410–18.795	0.021–0.090
	450 rpm_6 h_2 g	2.993–13.921	0.039–0.112
	450 rpm_12 h_2 g	1.788–22.481	0.039–0.147
Mass loading	450 rpm_6 h_1 g	0.977–13.687	0.031–0.134
	450 rpm_6 h_2 g	2.993–13.921	0.039–0.112
	450 rpm_6 h_3 g	2.028–25.108	0.026–0.140

**Fig. 3:**
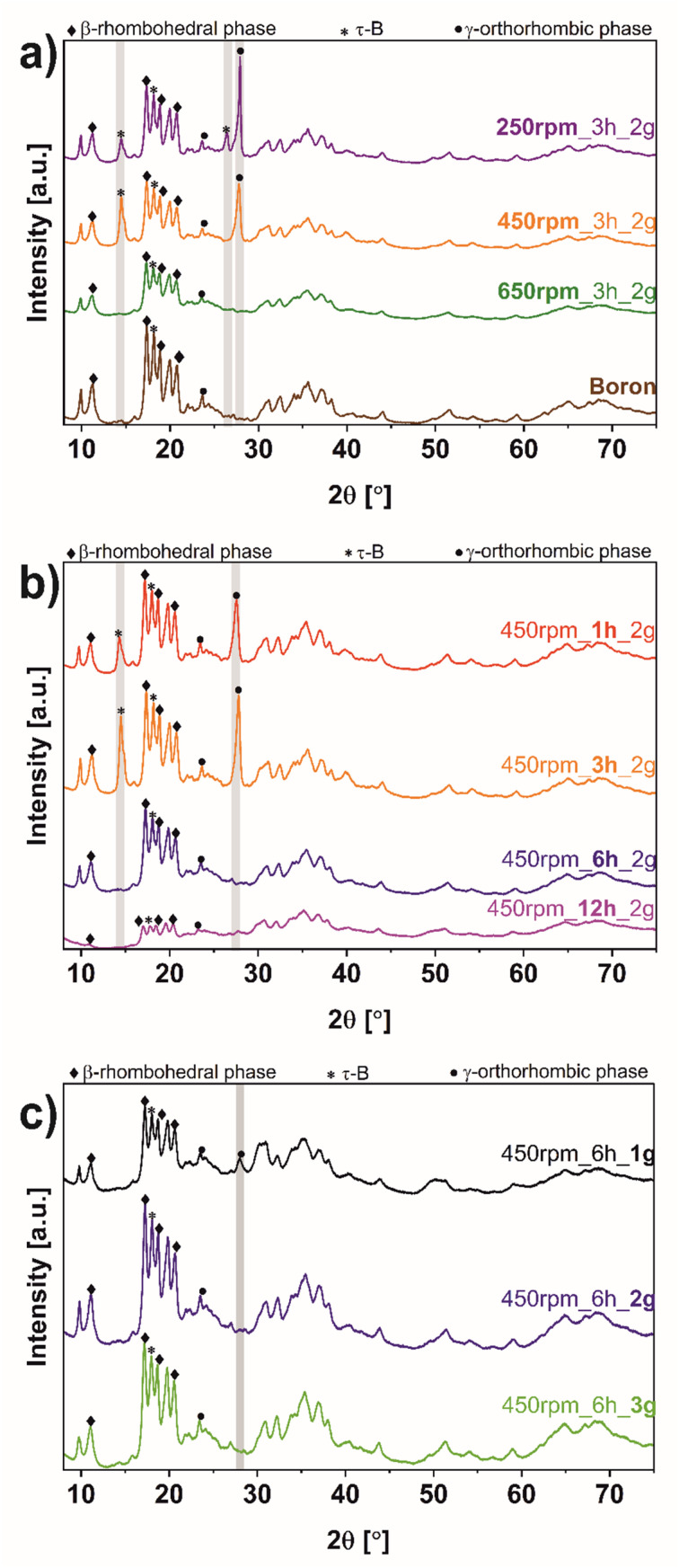
X-ray diffraction (XRD) of bulk boron and borophene flakes after the ball-milling process comparing (a) rotation speed, (b) time, and (c) mass loading.

Fig. 3 shows the XRD diffractograms of boron before and after the ball-milling process. For clear comparison the following sequence of parameters: rotation speed (Fig. 3a), time (Fig. 3b), and mass loading (Fig. 3c) were utilized. In all samples, characteristic reflections of bulk boron were noticed. To be more specific, the peaks at 11.1, 17.5, 19.0 and 20.8° correspond to the 003, 104, 021, and 015 respectively, correlate with β-rhombohedral structure, according to ICDD: 04-009-8808. The transformed additional phase based on the orthorhombic space group *Cmcm*, labeled as τ-B,^32^ is revealed in each sample at 18.3° (113). Additionally, a peak at 23.5° (110) is assigned to a γ-orthorhombic structure based on the pattern of ICDD: 01-078-2999. The structural changes and increased crystallinity have been indicated after the exfoliation process upon the ball-milling process. In the samples, 250 rpm_3 h_2 g, 450 rpm_3 h_2 g, 450 rpm_1 h_2 g, and 450 rpm_6 h_1 g, an additional peak at 27.9° (002) can be observed. This peak is attributed to the γ-orthorhombic structure (ICDD: 04-017-7208), compatible with the space group *Pnnm*, characteristic of borophene. Interestingly, more peaks from the τ-B structure appear after the ball-milling process in 250 rpm_3 h_2 g, 450 rpm_3 h_2 g, and 450 rpm_1 h_2 g at 14.5° (112) and only in 250 rpm_3 h_2 g at 26.5° (130) (ICDD: 04-023-0529). Suggesting that the formation of the τ-B structure is induced by rotation speed equal to 250 and 450 rpm, time of ball-milling 3 or 1 hour, and lower than 3 grams of mass loading. Moreover, considering the rotation speed (Fig. 3a), the reflections at 14.5, 26.5, and 27.9° have disappeared at 650 rpm (650 rpm_3 h_2 g). Thus, it can be concluded that reagglomeration at higher rotation speed occurs, which was also noticed by AFM analysis (Fig. 1). In the case of time of ball-milling (Fig. 3b) the peaks at 14.5 and 27.9° in 450 rpm_6 h_2 g and 450 rpm_12 h_2 g are observed, proving that an increase in the time of ball-milling up to 6 h causes reagglomeration of borophene flakes. The new diffraction at 27.9°, related to new crystalline phase formation, is only revealed at 450 rpm_6 h_1 g (Fig. 3c). Using a larger mass loading of bulk boron (450 rpm_6 h_2 g and 450 rpm_6 h_3 g) resulted in the disappearance of these reflections. Thus, it can be concluded, that increasing the loading weight makes the exfoliation process less efficient. Ball milling process parameters were optimized in respect to exfoliation efficiency and phase formation.

**Fig. 4:**
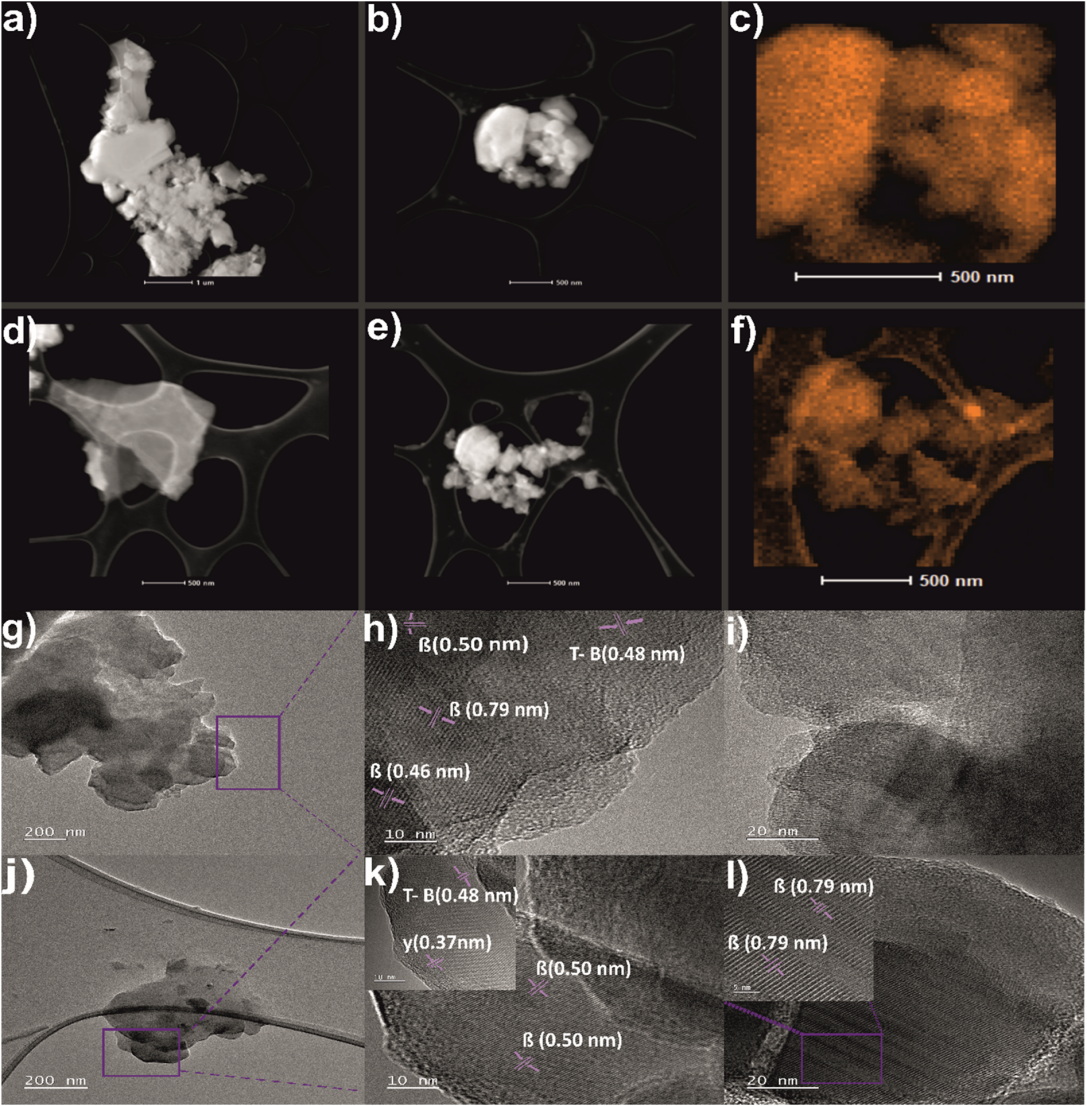
STEM with corresponding elemental mapping EDX and TEM images of studied materials: (a and b) STEM of bulk boron, (c) elemental mapping of boron on bulk boron, (d–e) STEM of borophene flakes after the ball-milling process [450 rpm_6 h_1 g], (f) elemental mapping of boron on borophene [450 rpm_6 h_1 g], (g–i) TEM images of boron, (j–l) TEM images of borophene flakes after the ball-milling process [450 rpm_6 h_1 g].

To investigate the structure and morphology of boron and borophene flakes the images by STEM with corresponding elemental mapping EDX and TEM, have been performed and shown in Fig. 4. It can be observed that densely packed bulk boron (Fig. 4a, b and g) exfoliates into thinner flakes of borophene flakes (Fig. 4d, e and j) when the ball-milling process occurs. Fig. 4c and f indicate the presence of elemental boron on the surface of bulk boron and borophene, respectively. The boron signal is more intense in the case of bulk which can originate from denser particles. Fig. 4g–l determines the flake-like structure of pristine boron and ball-milled borophene. Moreover, the *d*-spacings of both samples were calculated. In Fig. 4h, the *d*-spacing value calculated for bulk boron was approximately 0.46, 0.50, and 0.79 nm, which belongs to the 021, 104, and 003 planes of β-rhombohedral phase, respectively (ICDD: 04-009-8808). Moreover, Fig. 4i reveals the semi-crystalline character of bulk boron. The inter-lattice distance is equal to 0.48 nm which corresponds to 113 phases of τ-B, which has been detected in pristine boron (Fig. 4h) and after exfoliation (see inset of Fig. 4k) according to ICDD: 04-023-0529. Additional antilattice spacings equal to 0.50 and 0.79 nm are presented in the sample after the ball-milling process (Fig. 4k and inset 4l), confirming the presence of crystal phase β-rhombohedral one. Finally, the inset of Fig. 4k shows an interplanar distance of 0.37 nm attributed to 110 of the γ-orthorhombic phase present in the, a single-atom thick sheet of boron, what is in full agreement with XRD studies. Surface characterization of bulk boron and borophene flakes [450 rpm_6 h_1 g] by using a scanning electron microscope (SEM) was featured in the ESI Fig. S5.†

**Fig. 5:**
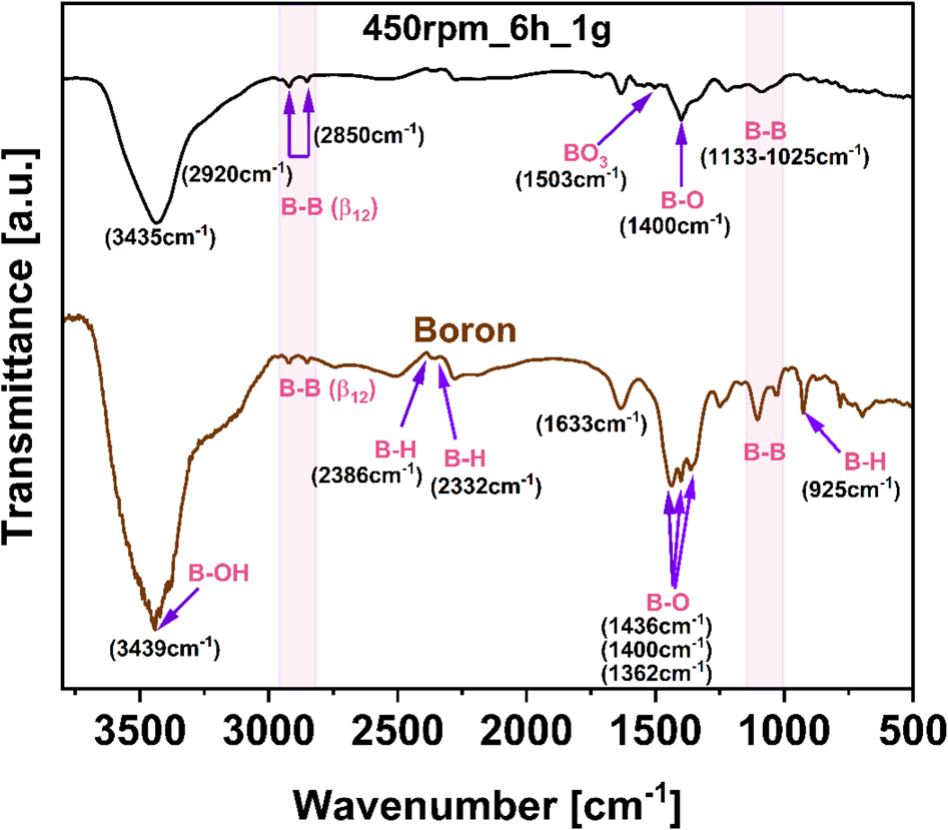
Fourier-transform infrared spectroscopy (FTIR) spectra of bulk boron and borophene flakes (450 rpm_6 h_1 g).

Regarding the chemical bonds and functional groups on the surface of the pristine boron and borophene flakes induced during the ball-milling process (450 rpm_6 h_1 g), FTIR analysis has been carried out (Fig. 5). Bulk boron presented a spectrum with sharper and more pronounced peaks compared with the spectrum of borophene (450 rpm_6 h_1 g). Observed less intense peaks in borophene were a consequence of the combined effect of shear and compression forces during the ball-milling process, which contribute to disrupting the chemical structure and defects formation.^33^ Giving more details, the band at 925 cm^−1^ in the spectrum of bulk boron is characteristic of B–H stretching. The mentioned above stretching is not visible in the spectrum of borophene. The mode 1025–1133 cm^−1^ corresponding to B–B stretching, is less intense and broader in borophene. The bond around 1362–1436 cm^−1^ has been attributed to B–O stretching and is attributed to the oxy-functional groups.^34^ It occurs in bulk boron and borophene (450 rpm_6 h_1 g), whereas this band broadens in 450 rpm_6 g_1 g, due to induced shear forces on energy relaxation and bonding affecting the vibration energy of atoms.^35^ The spectra of sample 450 rpm_6 h_1 g exhibited an additional band at about 1503 cm^−1^ as the vibrations of the bending mode of BO_3_ which is part of the structure of the B_2_O_3_ unit.^36^ Thus, after the ball-milling process, an increased amount of oxygen groups in studied material is noticeable. In both studied materials adsorbed moisture is indicated by water bonding at 1633 cm^−1^. Additionally, the peaks of B–H vibrations can be observed at 2332 and 2386 cm^−1^. The modes at 2850 and 2920 cm^−1^, observed in both materials, are attributed to characteristic binding B–B (β_12_).^37^ Additionally, the broad peak at 3435 cm^−1^, is assigned to B–OH stretching. The FTIR analysis confirmed that the ball-milling of boron induces the phase composition and chemical bonding modification as a result of having been modified by mechanical energy induced in the process.

**Fig. 6:**
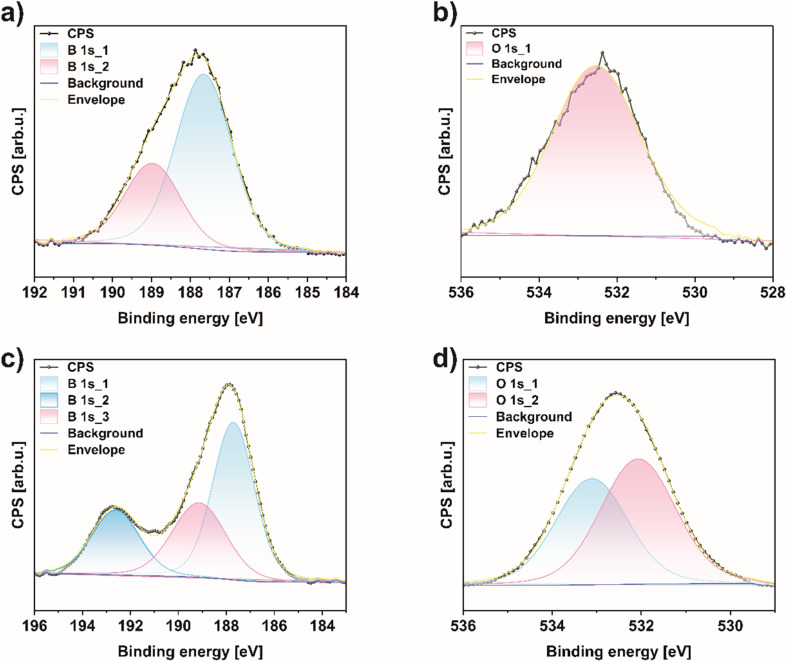
X-ray photoelectron spectroscopy (XPS) spectra of (a) B 1s, (b) O 1s region of bulk boron and (c) B 1s, (d) O 1s region of 450 rpm_6 h_1 g.

For a deeper understanding of the chemical composition and bonding of bulk boron and the obtained borophene (450 rpm_6 h_1 g), X-ray photoelectron spectroscopy (XPS) was conducted (Fig. 6). This provides insight into chemical changes in the B 1s and O 1s regions before and after the ball-milling process in the studied material. The overall XPS spectrum proved that the surface of 450 rpm_6 h_1 g mainly comprises B, C, N, and O elements (Fig. S6†). High-resolution scans in the B 1s in Fig. 6a and c exhibit two peaks around 189.1 and 187.6 eV for bulk boron and exfoliated few-layered borophene, corresponding to B–O and B–B bonding, respectively.^22^ What is more, after the ball-milling process, a new signal at 192.5 eV emerges and is attributed to the existence of B_2_O_3_ in the sample 450 rpm_6 h_1 g, presented in Fig. 6c. In addition, the content of these bonds in borophene are 22.7, 29.3, and 48.0% for B_2_O_3_, B–O, and B–B, correspondingly, which proves that borophene is more oxidized in respect to bulk boron.^38^ These results are in agreement to FTIR analysis presented above. For a deeper understanding, quantitative analyses of the above-mentioned bindings in both samples are reported and shown in Table 2. Additionally, the O 1s region in bulk boron was deconvoluted only in one peak at 532.08 eV correlated with C–OH bonding (Fig. 6b).^39^ On the other hand, this region in 450 rpm_6 h_1 g shows two peaks at 533.08 and 532.08 eV attributed to C–O, and C–OH bonding, respectively (Fig. 6d).

**Table 2:** XPS data were obtained for bulk boron and borophene after the ball-milling process

	Composition [%]		
Sample	B_2_O_3_	B–O	B–B
Bulk boron	—	22.97%	77.03%
Borophene [450_6 h_1 g]	22.70%	29.30%	48.00%

## 5. Conclusions

A simple and scalable technique such as the ball-milling process for obtaining exfoliated borophene flakes was successfully performed. The morphology of exfoliated bulk boron under different operating conditions such as rotation speed (250–650 rpm), time of ball-milling (1–12 hours), and mass loading (1–3 g) was investigated. An atomic force microscope (AFM) showed changes in the thickness and size of the borophene materials after the ball-milling process. What is more, pointed that borophene 450 rpm_6 h_1 g was the thinness sample (∼5.5 nm). It can be concluded that the selection of appropriate parameters in ball-milling conditions can be simplistic to fabricate a few-layer borophene. Moreover, it has been proven that the ball-milling process related to the new crystal phases formation. The results showed a content of β-rhombohedral, γ-orthorhombic, and τ-B phase structures in borophene. On the other hand, a thorough X-ray diffraction (XRD) analysis indicates that the formation of new phases depends on the adopted operational parameters. To a greater extent further used borophene, obtain in this direct way, gives considerable possibilities for hands-on application and modification.

## Conflicts of interest

There are no conflicts to declare.

## Supplementary Material

RA-013-D3RA02400H-s001
