# Supplementary material for: Ball milling induced borophene flakes fabrication
Source: RSC Adv. 2023 Jun 5;13(25):16907–14. doi: 10.1039/d3ra02400h (PMC10241196; doi:10.1039/d3ra02400h)
Supplement: RA-013-D3RA02400H-s001 [file RA-013-D3RA02400H-s001.pdf]

Electronic Supplementary Material (ESI) for RSC Advances.
This journal is © The Royal Society of Chemistry 2023

Supporting information

Ball milling-induced borophene flakes fabrication

Klaudia Zielinkiewicz*, Daria Baranowska, Ewa Mijowska**

Department of Nanomaterials Physicochemistry, Faculty of Chemical Technology and Engineering, West Pomeranian University of Technology, Szczecin, Piastow Ave. 42, 71-065 Szczecin, Poland

Corresponding author: *zk43130@zut.edu.pl; **emijowska@zut.edu.pl

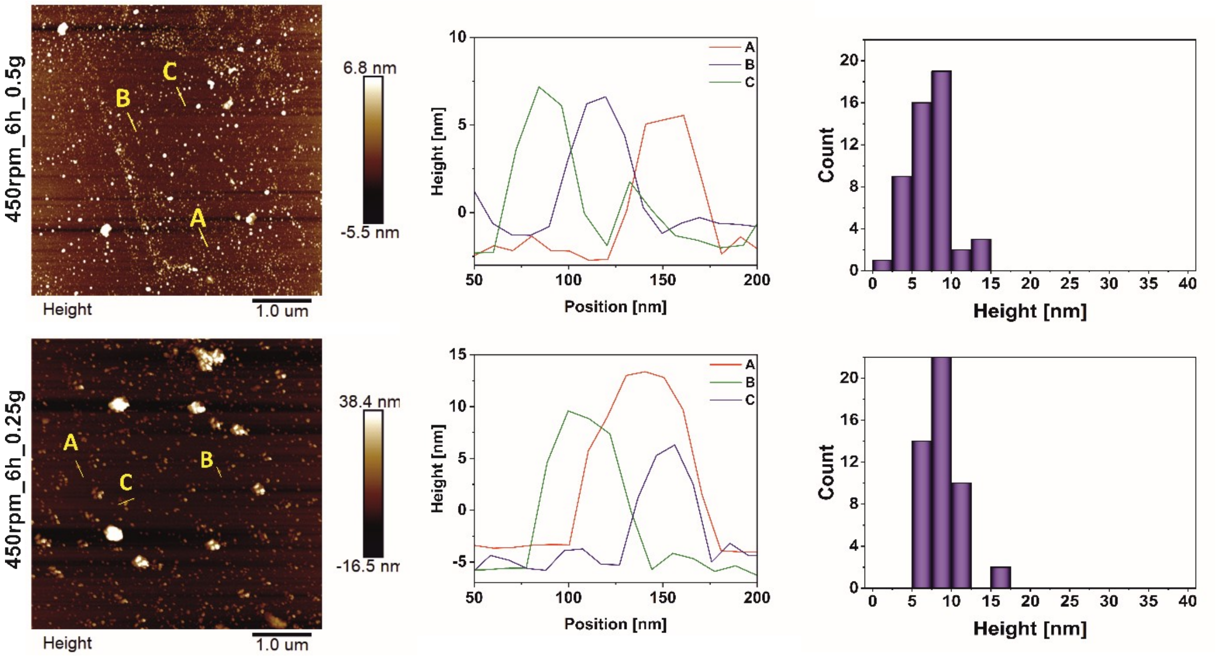

Figure S1. *Atomic force microscopy (AFM) images of samples 450_6h_0.5g and 450rpm_6h_0.25g (left panel) and height profiles of representative borophene flakes named A, B, C (middle panel) and corresponding histograms presenting thickness distribution of respective sample (right panel).*

Table 1. The statistical distribution of the thickness and lateral size.

Sample	Thickness [nm]	Lateral size [µm]
450rpm_6h_0.5g	2.021-14.439	0.031-0.387
450rpm_6h_0.25g	5.544-15.798	0.030-0.660

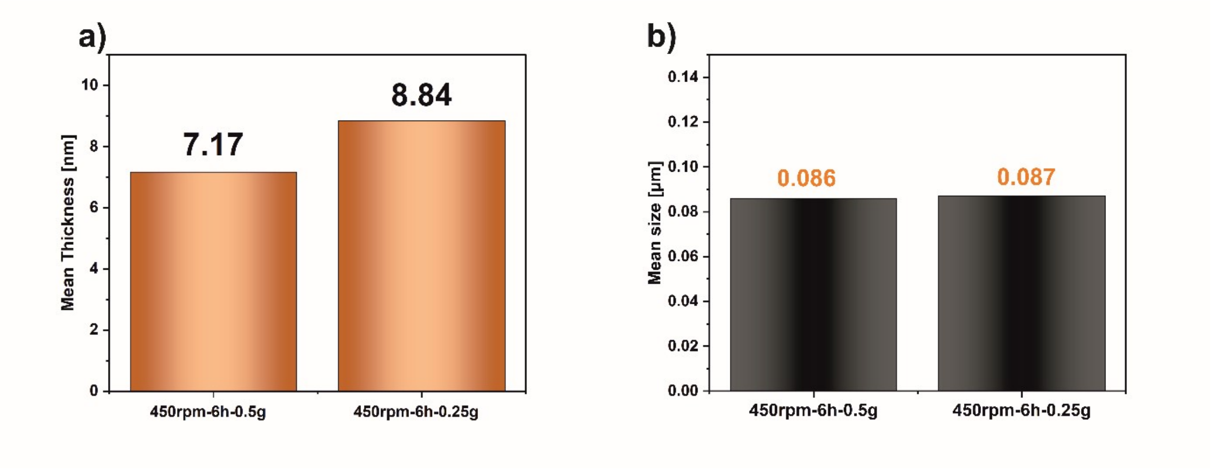

Figure S2. *Statistical distribution of a) mean thickness and b) mean size of the samples [450rpm_6h_0.5g and 450rpm_6h_0.25g].*

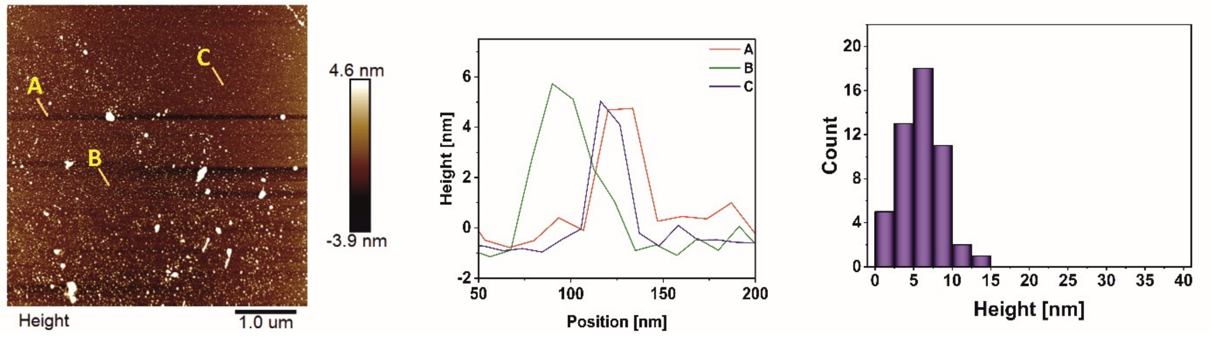

Figure S3. *Atomic force microscopy (AFM) image of sample 550rpm_6h_1g (left panel) and height profiles of representative borophene flakes named A, B, C (middle panel) and corresponding histograms presenting thickness distribution of respective sample (right panel).*

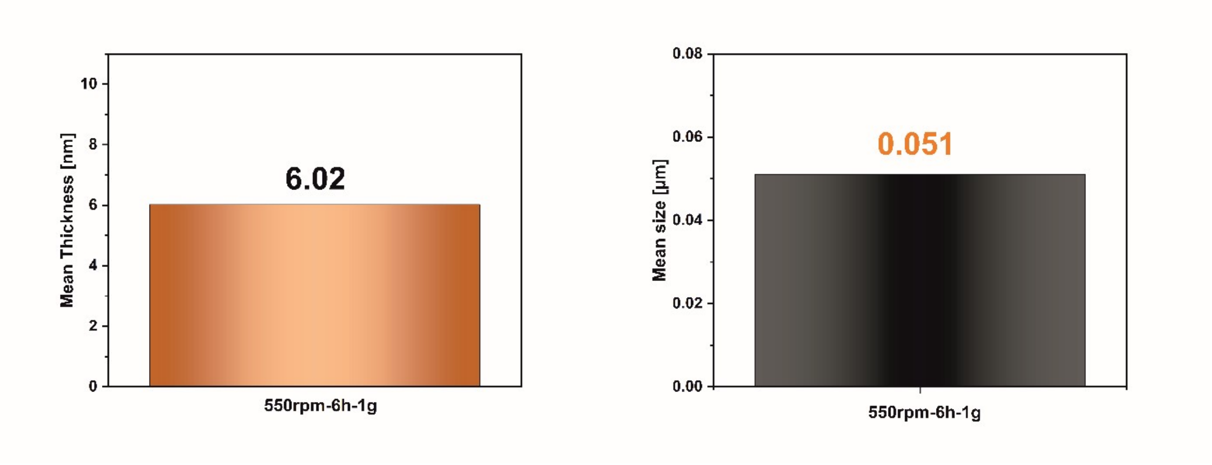

Figure S4. *Statistical distribution of a) mean thickness and b) mean size of the sample [550rpm_6h_1g].*

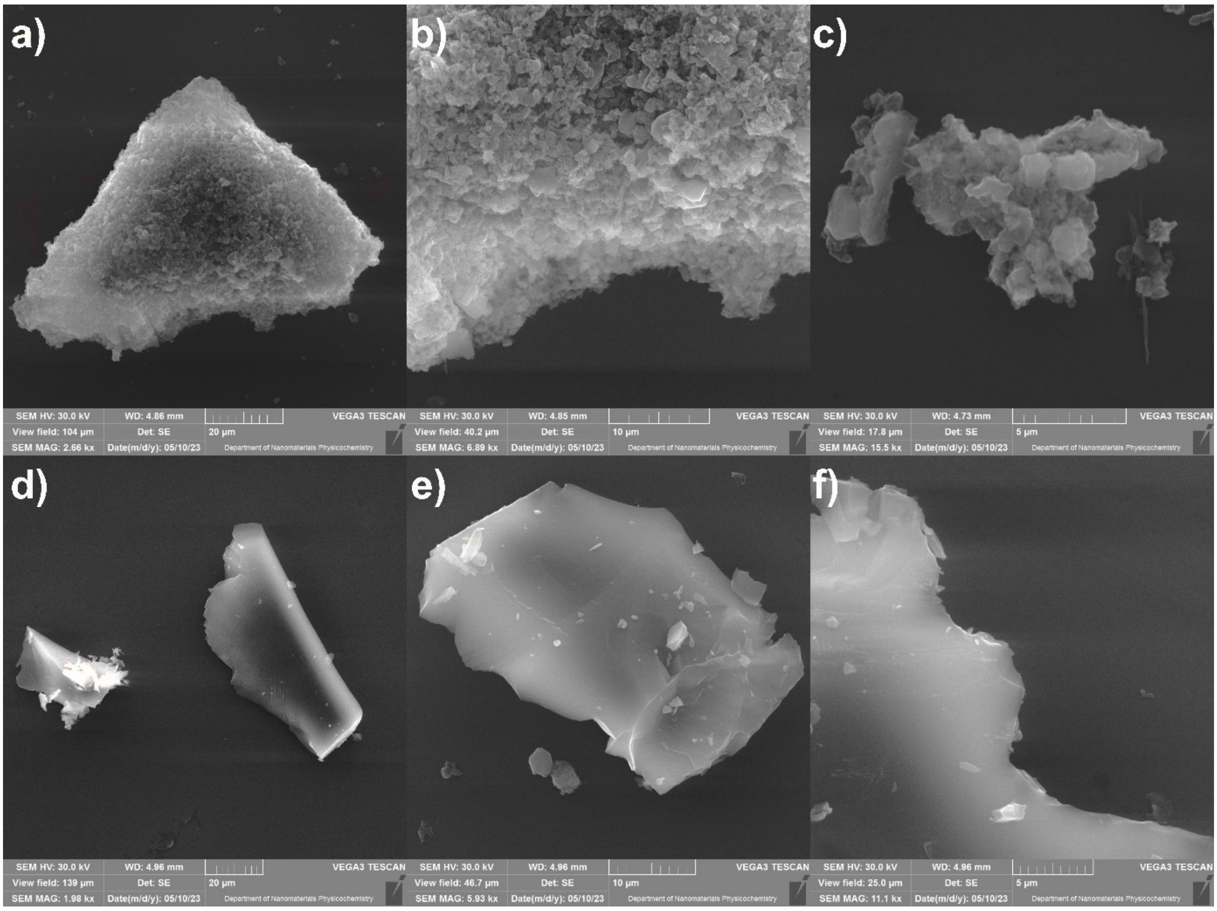

Figure S5*. SEM images of (a-c) bulk boron, (d-f) borophene [450rpm_6h_1g].*

Figure S5 shows a scanning electron microscope (SEM) of the surface morphology of bulk boron (**Figure S5a-b**) and studied material after the ball-milling process (**Figure S5d-f**) under

optimal operating conditions 450 revolutions per minute, 6 hours, and 1g of a batch. All SEM images present flake-like structure particles of boron and borophene flakes. Significant morphological changes have been noted. It found that bulk boron exhibits an unclear and densely packed structure to compare with well-developed surface of the exfoliated sample. **Figures S5a-c** macroscopically proved that using different parameters of the ball-milling process the changes in the structure of boron occured. Agglomeration phenomena are clear in bulk boron in respect to borophene after exfoliation. This reveals that the exfoliation process was successful. The differences in thickness are noticeable through approaching the edge of boron (**Figure S5b**) and borophene (**Figure S5f**). In the first case, many unevennesses and superimposed layers are visible, while exfoliated boron displays a clearly outlined shape. This phenomenon has also been confirmed using techniques such as TEM and AFM.

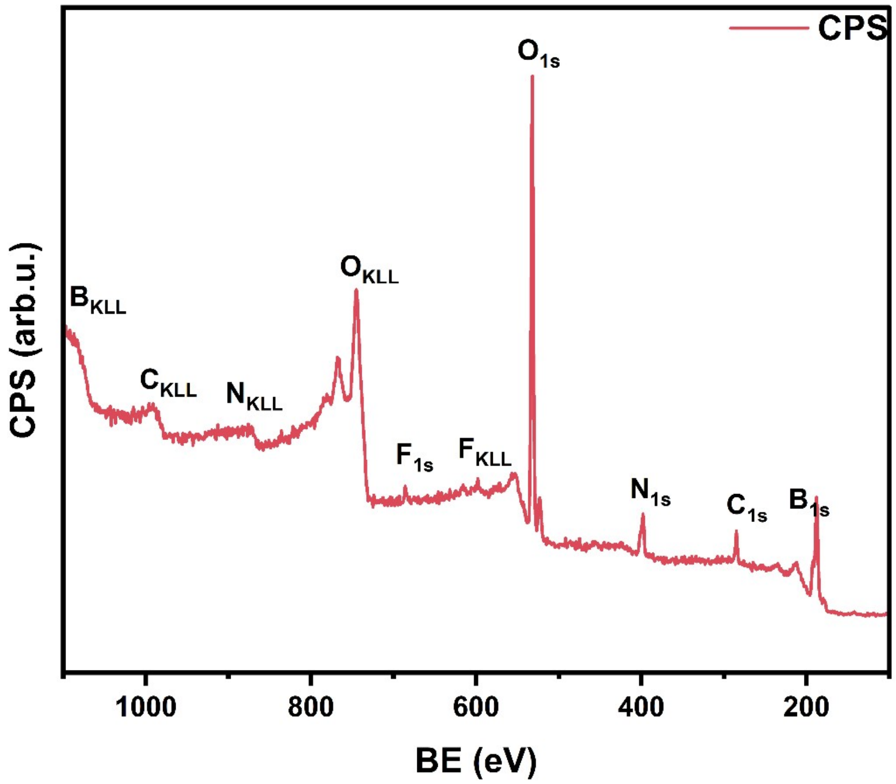

***Figure S6**. Survey XPS spectrum of borophene sample 450rpm_6h_1g.*
